# Metal–organic frameworks for electronics: emerging second order nonlinear optical and dielectric materials

**DOI:** 10.1088/1468-6996/16/5/054204

**Published:** 2015-10-06

**Authors:** Shruti Mendiratta, Cheng-Hua Lee, Muhammad Usman, Kuang-Lieh Lu

**Affiliations:** Institute of Chemistry, Academia Sinica, Taipei 115, Taiwan

**Keywords:** metal–organic frameworks, nonlinear optical, dielectric

## Abstract

Metal–organic frameworks (MOFs) have been intensively studied over the past decade because they represent a new category of hybrid inorganic–organic materials with extensive surface areas, ultrahigh porosity, along with the extraordinary tailorability of structure, shape and dimensions. In this highlight, we summarize the current state of MOF research and report on structure–property relationships for nonlinear optical (NLO) and dielectric applications. We focus on the design principles and structural elements needed to develop potential NLO and low dielectric (low-*κ*) MOFs with an emphasis on enhancing material performance. In addition, we highlight experimental evidence for the design of devices for low-dielectric applications. These results motivate us to develop better low-dielectric and NLO materials and to perform in-depth studies related to deposition techniques, patterning and the mechanical performance of these materials in the future.

## 1. Introduction

Metal–organic frameworks (MOFs) have emerged in the past few years as a promising class of materials with a wide spectrum of useful applications [1–3]. Their unique properties arising from the self-assembly of metal ions/clusters with electron-donating organic linkers, enables ordered frameworks with high uniform porosities, ultralow densities and high thermal stabilities to be created [4, 5]. Their tunable pore sizes, rich coordination chemistry and fascinating topologies provide a suitable platform to a wide array of potential applications that include host−guest interactions, such as gas storage, sensing, chemical separations and catalysis [6–11]. In comparison with these applications, significantly less effort has been directed towards the incorporation of MOFs in optical and microelectronic devices.

**Figure 1.**
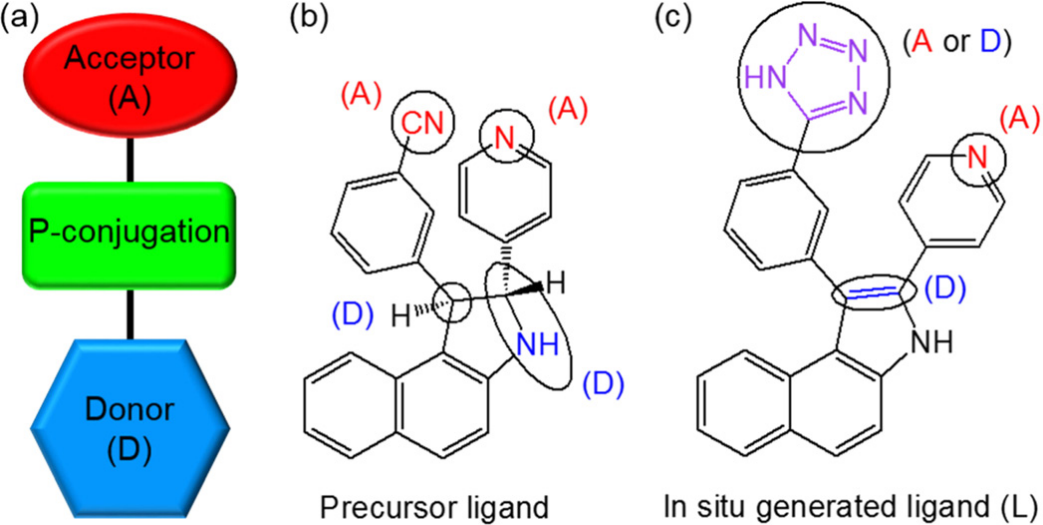
(a) Schematic illustration of a donor–acceptor system; (b) ligand reported by Xiong and coworkers [25, 26]; (c) the corresponding *in situ* generated ligand.

**Figure 2.**
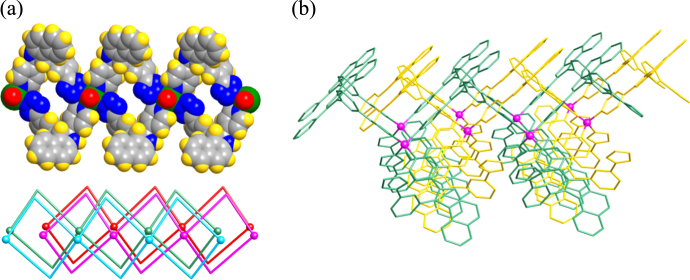
Compounds reported by Xiong and coworkers [25, 26] (a) 1D coordination polymer with the formula [Cd(H_2_tzpbin)_2_(H_2_O)_2_]_*n*_; (b) 2D coordination polymer [Zn(tzpbin)_2_]·1.5H_2_O.

In recent years, nonlinear optical (NLO) materials have been identified as promising candidates for emerging photonic devices such as high-speed optical modulators, ultra-fast optical switches and high-density optical data storage based on the fact that photons are capable of processing information at the speed of light [12–15]. On the other hand, the number of active components in an integrated circuit has been exponentially increasing with increasing miniaturization of electronic devices, doubling approximately every two years [16]. The corresponding decrease in transistor size brings with it other issues such as increased resistance of the interconnect wires, propagation delay and crosstalk between the wires. These problems can be eliminated by the introduction of a new interlayer low-*κ* dielectric material [17]. Current theoretical and experimental results regarding NLO and low-*κ* MOFs highlight the potential use of MOFs as new materials for photonics and microelectronics [18]. The objective of this article is to highlight recent developments and attempts to use MOFs as NLO and dielectric materials and establishing the structure–property relationships required to enhance material performance.

## 2. NLO materials for photonics

It is crucial to develop an understanding of the tools used to manipulate photons before discussing NLO technologies. Materials need to be synthesized that are capable of amplifying a light signal, alter the frequency of light, modulate phase factors and light intensity. Through second-harmonic generation (SHG), a NLO material facilitates the ‘adding-up’ of two photons to form a new one with twice the frequency. In theory, the SHG intensity *I*_2*ω*_ from any interface of the crystal in either reflection or transmission geometry is proportional to the square of the NLO coefficient *χ*^(2)^ and to the energy of the fundamental frequency beam *I*_*ω*_ (equation (1))

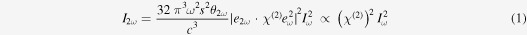
where*θ* is the angle from the surface normal, at which the SHG signal occurs, the vectors*e*_*ω*_ and*e*_*2ω*_ describe the fundamental and the second harmonic light fields at the surface [19]. Only materials crystallizing in non-centrosymmetric crystal classes can have a non-vanishing*χ*^(2)^. Before the kick start of research on organic polymers and MOFs for NLO applications, the major interest was focused on non-centrosymmetric inorganic materials, such as quartz, potassium dihydrogen phosphate (KDP = KH_2_PO_4_),*β*-barium borate and semiconductors such as cadmium sulfide and tellurium [20,21]. A lack of extended*π*-electron delocalization, difficulties associated with the synthesis, their low optical quality resulted in pure inorganic materials typically having low NLO responses and slow response times. This opened the doors for organic compounds such as urea crystals which in recent years became a SHG standard because of its high SHG efficiency, good optical damage threshold and its abundance [20]. The second-order NLO susceptibility*χ*^(2)^ is related to the first hyperpolarizability*β* of a molecule, where*β* is influenced by transition moments and the dipole moment difference between the ground and charge transfer excited state [22]. Conjugated molecules that contain a donor–acceptor system have a large transition state and excited state dipole moments. However, majority of organic conjugated molecules form centrosymmetric structures because of dipole–dipole interactions [13,23]. In addition, drawbacks such as poor mechanical strength and low physicochemical stability restrict them for device incorporation [12].

**Figure 3.**
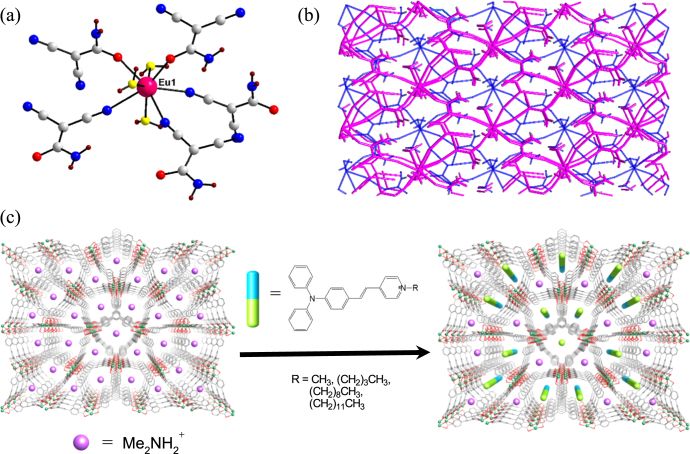
(a) Coordination mode of Eu(III) center in {[Eu(cda)_3_(H_2_O)_3_]·H_2_O}_∞_ (cda = carbamyldicyanomethanide); (b) 2D framework in {[Eu(cda)_3_(H_2_O)_3_]·H_2_O}_∞_ with (4,4) net topology (SHG value of 16.8 × urea); (c) schematic illustration of pyridinium hemicyanine chromophores incorporated into ZJU-28. (Me = methyl.)

In the last decade, considerable emphasis has been laid on the rational design and synthesis of non-centrosymmetric MOFs, which can be tailored to contain well-defined pores and to have a rich coordination chemistry. Judicious synthetic approaches for developing MOFs with non-centrosymmetric linkers were reviewed by Lin*et al* [13,23]. Lin and coworkers also reported, for the first time, on the NLO properties of chiral and acentric 2D square grids, bis(nicotinato)zinc and bis-cadmium, synthesized using bifunctional bridging ligands,*m*-pyridinecarboxylates, as linking groups [24]. Their Cd-based compound exhibited a powder SHG efficiency larger than technologically important LiNbO_3_ (*I*_2*ω*_ of 1000 versus*α*-quartz). Xiong and coworkers reported a 1D Cd-based coordination polymer [Cd(H_2_tzpbin)_2_(H_2_O)_2_]_*n*_ (H_3_tzpbin =*trans*-2,3-dihydro-2-(4′-pyridyl)-3-(3′-cyanophenyl)benzo[*e*]indole) which displayed an impressive SHG response (figure2(a)) in the powdered state (80 × urea) [25]. The compound was synthesized using a ligand with an excellent two-chiral center-A-D chromophore, essential for achieving second-order optical nonlinearity (figure1). The cyano group present on the ligand, could be readily converted into a tetrazole functionality in the presence of NaN_3_ and*in situ* generated a chiral tetrazole ligand H_3_tzpbin (figures1(b) and (c)). The molecule with two chiral centers, lacked inversion symmetry and a mirror plane, and crystallized in non-centrosymmetric space group. The strong enhancement in SHG response may be attributed to the synergistic effect exerted by the highly asymmetric nature of the ligand induced by the two functional electronic asymmetry (or the push–pull effect) and metal–ligand coordination. This type of high SHG response was also exhibited by the Zn-based compound, a 2D coordination polymer [Zn(tzpbin)_2_]·1.5H_2_O (figure2(b)), synthesized by Xiong and coworkers using the same ligand and showing the second highest SHG intensity (50 × urea) [26]. In addition, Qian and coworkers developed an effective strategy for incorporating dipolar organic chromophores such as 4-(4-(diphenylamino)styryl)-1-dodecylpyridinium bromide (DPASD) into (Figure3(c)) the one-dimensional channels of an anionic porous MOF (ZJU-28), to generate highly active NLO materials [27]. These results open new possibilities for the development of promising NLO active MOFs.

Although many MOFs have been reported for NLO applications, including those synthesized by us [28,29], however, we show a selected list of those with the highest reported SHG intensity values in table1. From the published literature, the future of MOF’s as the next generation of NLO materials is obvious. Therefore, to achieve a high NLO response, non-centrosymmetric organization of the framework is necessary, which can be ensured by introducing aromatic chiral ligands or unsymmetrical ligands exerting push–pull effects (electron donor–acceptor system). Secondly, it has been reported that the incorporation of alkali and alkaline earth ions into the Cd^2+^/carboxylate frameworks may greatly enhance the SHG response in acentric MOFs. Finally, large SHG values may also be possible by encapsulating matching ordered dipolar chromophores in the pores of porous MOFs.

**Table 1.** Highest reported SHG response for metal–organic frameworks.

MOFs	NLO	References
[Cd(H_2_tzpbin)_2_(H_2_O)_2_]_*n*_	80 × urea	[25]
[Zn(tzpbin)_2_]·1.5H_2_O	50 × urea	[26]
{[Eu(cda)_3_(H_2_O)_3_]·H_2_O}_∞_	16.8 × urea	[30]
[Mn(Hdnty)_2_]	6 × urea	[31]
[Nd(Hdnty)_2_(NO_3_)(H_2_O)_5_]·3H_2_O	5 × urea	[31]
Zn(aptz)_2_	5 × urea	[26]
[Ag(bcdc)]ClO_4_	2.9 × urea	[32]
[Zn(lac)(nic)]	1.2 × urea	[33]
Zn(ptz)_2_	1 × urea	[26]
{[Cd_2_(dpys)(D-cam)_2_(H_2_O)_2_]·H_2_O}_*n*_	0.8 × urea	[34]
[Zn(1,3-bimb)(D-cam)]_*n*_	0.3 × urea	[34]
[Zn(1,4-bimb)(D-cam)]_*n*_	0.3 × urea	[34]
[Cu(bcdc)]PF_6_·THF	0.2 × urea	[32]

H_3_tzpbin = (1R,2R)-1-(3-(1H-tetrazol-5-yl)phenyl)-2-(pyridin-4-yl)-2,3-dihydro-1H-benzo[e]indole; cda = carbamyldicyanomethanide; H_2_dnty = 3,5-dinitrotyrosine; aptz = 5-(6-aminopyridin-3-yl)tetrazol-1-ide; bcdc = N,N′-bis(4-cyanophenyl)-(1R,2R)-diaminocyclohexane; lac = ethyl S-lactate; nic = isonicotinate; ptz = 5-phenyl tetrazolate; dpys = 4,4′-dipyridylsulfide; D-cam = D-camphorate; 1,3-bimb = 1,3-bis(imidazol-1-ylmethyl)benzene; 1,4-bimb = 1,4-bis(imidazol-1-ylmethyl)benzene; THF = tetrahydrofuran.

## 3. Low-*κ* materials for microelectronics

**Figure 4.**
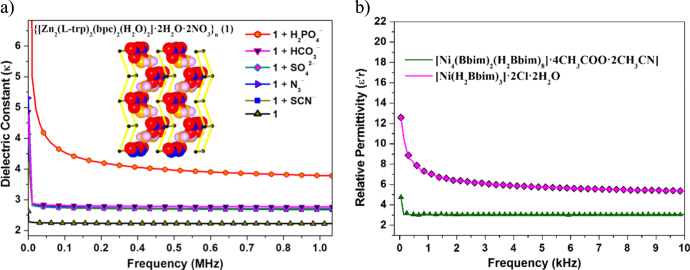
(a) Schematic illustration of dielectric properties of Zn-based chiral MOF influenced by the anions. (b) Comparison of permittivity values of Ni-based supramolecular compounds incorporating different solvent molecules.

As per the International Technology Roadmap for Semiconductors, porous materials and air gap structures promise to become the future low-*κ* materials by replacing SiO_2_ in inter-layer dielectrics (ILDs), thereby substantially reducing interconnect resistance/capacitance delay and the crosstalk noise in ICs^1^ 1See 2011 Ed. The International Technology Roadmap for Semiconductors (ITRS). MOFs have been proposed as possible candidates for ILDs since they are highly thermally stable at temperatures of up to 400 °C, show electrically insulating behavior, high mechanical strength and chemical stability [35]. Since the time Hermann and coworkers reported a brief simulated proof for the use of MOFs as low-*κ* materials [18], especially IRMOF-M2c [Zn_4_O(tdca)_3_] (tdca = tetracene-2, 8-dicaboxylate) with a calculated*κ* value as low as 1.21, only a few groups (including ours) have experimentally determined the dielectric properties of MOFs. Since the dielectric constant (*κ*) is directly related to the polarization mechanism, a higher polarization implies an increase in the dielectric constant [17]. We envisaged that the removal of the polar guest molecules from the framework and increasing the porosity of the framework would decrease the polarization and, hence, the dielectric constant (*κ*). To develop a more in-depth understanding of the factors that influence dielectric properties, we studied various conditions including the influence of various constituents such as anions, solvents and organic linkers for fine tuning and optimizing the dielectric constant. We reported on the anion-controlled dielectric behavior of amino acid-based two-dimensional homochiral MOFs (figure4(a)). They are found to be isostructural. The Zn derivative [{[Zn_2_(L-trp)_2_(bpe)_2_(H_2_O)_2_]·2H_2_O·2NO_3_}_*n*_ (**1**, L-Htrp = L-tryptophan, bpe = 1,2-bis(4-pyridyl)ethylene)] showed lower dielectric constants (*κ*) than the Co one. The dielectric values of the Zn species were influenced by the type of exchanged anions [29].

Furthermore, we reported on the nickel (II)-based supramolecular compounds [36] which showed a significant reduction in*κ* value (figure4(b)) when highly polarizable guest molecules (Cl^−^, H_2_O;*κ* = 5.36 at 10 kHz) were replaced by less polarizable ones (CH_3_COO^−^, CH_3_CN;*κ* = 3.03 at 10 kHz). We further examined and restricted the polar guest molecules from occupying the pores of 3D MOFs using hydrophobic ligands. Our Mg-based MOF, [Mg(phen)(bdc)]_*n*_ (phen = 1,10-phenanthroline, bdc^2−^ =1,4-benzenedicarboxylate) was recently found to possess a solvent free framework, a non-interpenetrated network with an uncommon*cds*-type topology [37]. Chemical and dielectric investigations of this material indicated that it retained its high chemical stability and had a low dielectric constant of 3.3 ± 0.1 (at 100 kHz), the lowest value for a Mg-based MOF reported to date. In addition, temperature-dependent dielectric studies revealed that its low dielectric constant was retained over a wider temperature range. Although 1,10-phenanthroline was used in the above experiment, we believe that guest free stable frameworks could also be formed using similar ligands such as 4,7-phenanthroline and ligands containing naphthalene and anthracene moieties. Dielectric constant (*κ*) values for a few low-*κ* MOFs are shown in table2.

**Table 2.** Comparison of reported low-*κ* MOFs (*f* ≤ 0.1 MHz).

MOFs	*κ*	References
{(Zn(MeIM)_2_}^a^ (ZIF-8)	∼2.33	[38]
[Sr_2_(1,3-bdc)_2_]^b^	∼2.4	[39]
{[Pb(Tab)_2_(4,4′-bipy)](PF_6_)_2_·2MeCN}^c^	∼2.53	[40]
{[Zn_2_(L-trp)_2_(bpe)_2_(H_2_O)_2_]·2H_2_O·2NO_3_}_*n*_^d^	∼2.53	[29]
{Mn_2_(D-cam)_2_(2-Hpao)_4_}_*n*_^e^	∼2.8	[41]
{[Co_2_(D-cam)_2_-(3-abpt)_2_(H_2_O)_3_]_*n*_·5nH_2_O}^e^	∼3.0	[41]
{[Ni_2_(bbim)(H_2_bbim)_4_]·2CH_3_COO·CH_3_CN}_2_^f^	∼3.03	[36]
{[Pb(Tab)_2_]_2_(PF_6_)_4_]·2MeCN·DMF}^c^	∼3.04	[40]
{[Co(L-trp)(bpe)(H_2_O)]·H_2_O·NO_3_}_*n*_	∼3.30	[29]
[Mg(phen)(bdc)]_*n*_	∼3.33	[37]
{[(C_3_H_7_)_2_NH_2_][Cr_7_NiF_8_(O_2_C_4_H_5_)_16_]-MMA}	2.9 ∼ 5	[42]
(±)-[Ni(H_2_bbim)_3_]·2Cl·2H_2_O	∼5.36	[36]
[Zn(TMPT)_2_]_*n*_^g^	∼6	[43]

a MeIM = 2-methylimidazolate.

b 1,3-bdc = benzene-1,3-dicarboxylate.

c Tab = 4-(trimethylammonio)benzenethiolate, 4,4′-bipy = 4,4′-bipyridine.

d L-trp = L-tryptophanate, bpe = 1,2-bis(4-pyridyl)ethylene).

e D-cam = D-camphorate, 2-Hpao = 2-pyridinealdoxime, 3-abpt = 4-amino-3,5-bis(3-pyridyl)-1,2,4-triazolate.

f H_2_bbim = bisbenzimidazole.

g TMPT = 5-(4-((1H-1,2,4-triazol-1-yl)methyl)phenyl)-2H-tetrazolate; DMF = dimethylformamide; MMA = methyl methacrylate.

From this literature survey, the future of MOF’s as the next generation of low-*κ* materials is clear. To synthesize a potential low-*κ* MOF, the node and linker strategy could be applied to produce porous frameworks. To prevent or inhibit polar molecules from occupying the cavity, more hydrophobic ligands could be introduced and high dielectric solvents should be avoided during synthesis. In addition, if the pores become occupied by the polar guest molecules the sample should be dehydrated and activated, lastly to maintain the overall polarization low, hard and less polarizable anions based on hard–soft acid–base principle could be included. Through a judicious combination of theoretical calculations and fundamental studies a promising low-*κ* MOF which can potentially replace SiO_2_ in ILD applications could be generated. However, the integration of MOFs as components of functional devices is still in its initial stages of development. The fundamental issues associated with circuit design, leakage current, and thin film growth need to be acknowledged and resolved. The development of nano-sized MOFs and the deposition of MOF thin films represents a particularly crucial starting point for device design and fabrication. Liquid-phase growth methods have been the dominant process for the growth of MOF thin films reported to date [44]. Other related methods such as atomic-layer deposition, vapor-phase epitaxy and sputtering methods have never been used for MOFs until now and only a few MOFs can be grown on any type of surface.

**Figure 5.**
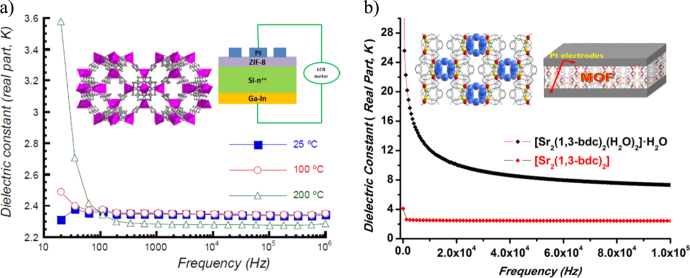
Dielectric measurements including device design and integration in (a) ZIF-8 films (reprinted with permission from*Chem. Mater.***25** 27, Copyright (2015) American Chemical Society); (b) Sr-based 3D-MOF {[Sr_2_(1,3-bdc)_2_(H_2_O)_2_]·H_2_O}_*n*_ and the corresponding MIM device. Inset shows a schematic of the MIM device.

The first experimental evidence for such a device was provided by Saiz and coworkers based on studies of ZIF-8 films which had an effective*κ* value of 2.4 (figure5(a)), necessary for future low-*κ* chips [38]. The films also showed good mechanical properties, hydrophobicity, and they outperformed other organic and inorganic low-*κ* candidates in terms of pore size and hydrophobicity. They constructed MIM (metal–insulator–metal)-type capacitors by depositing Pt dots on top of ZIF-8 films supported on silicon wafers (Inset in figure5(a)). Following the roadmap and taking inspiration from the above example, we reported on a new low-*κ* strontium-based MOF {[Sr_2_(1,3-bdc)_2_(H_2_O)_2_]·H_2_O}_*n*_ [39], which after dehydration, due to loss of polarization, showed a low dielectric value of 2.4 at 1 MHz (figure5(b)) with a very low dielectric loss (0.026). The dehydrated compound was also found to be thermally stable at temperatures up to 420 °C. In this case, the MIM device was constructed by depositing a Pt electrode layer with a thickness of 30 nm on both sides of the pellets of Sr-MOF (inset in figure5(b)). The measurements showed a low leakage current of 1.69 × 10^–9^ A mm^−2^, making it a potential candidate for use as an ILD device.

The dielectric and optical studies of MOFs are in its initial stages and further research will be needed to integrate them as active components in actual devices. The fabrication of MOFs into thin films has been reported as the starting point for introducing MOFs into real devices [44]. In addition, the fundamental properties of MOFs discussed here highlight the need for expanded research beyond synthesis to lithography, process design, and material integration.

## 4. Summary

To summarize, MOFs offer a unique combination of thermo-mechanical stability, synthetic flexibility and tunable structural properties. The incorporation of non-centrosymmetry in the structure creates the potential for their use in NLO, while the introduction of highly ordered porosity and the evacuation of polar guest molecules suggest that MOFs represent an ideal replacement as an ILD material. At the current stage, addressing the many issues related to device design and fabrication would be highly desirable. The development of generalized fabrication methods for use in growing MOF thin films and identifying other necessary materials required for device integration and completion, will enable MOFs to be used to the fullest extent of their properties. The foregoing results will provide an effective path for resolving the above issues and will create more opportunities in the exciting new subdiscipline in the field of MOFs.
